# Tumor-selective mitochondrial network collapse induced by atmospheric gas plasma-activated medium

**DOI:** 10.18632/oncotarget.7889

**Published:** 2016-03-03

**Authors:** Kosuke Saito, Tomohiko Asai, Kyoko Fujiwara, Junki Sahara, Haruhisa Koguchi, Noboru Fukuda, Miki Suzuki-Karasaki, Masayoshi Soma, Yoshihiro Suzuki-Karasaki

**Affiliations:** ^1^ Innovative Therapy Research Group, Nihon University Research Institute of Medical Science, Tokyo, Japan; ^2^ Division of General Medicine, Department of Medicine, Nihon University School of Medicine, Tokyo, Japan; ^3^ Department of Physics, College of Science and Technology, Nihon University, Tokyo, Japan; ^4^ The National Institute of Advanced Industrial Science and Technology, Tsukuba, Japan; ^5^ Division of Nephrology Hypertension and Endocrinology, Department of Medicine, Nihon University School of Medicine, Tokyo, Japan; ^6^ Department of Dermatology, Nihon University Surugadai Hospital, Saitama, Japan; ^7^ Division of Physiology, Department of Biomedical Sciences, Nihon University School of Medicine, Tokyo, Japan

**Keywords:** non-thermal atmospheric gas plasma (AGP), AGP-activated medium, mitochondrial network, reactive oxygen species (ROS), tumor-selective killing

## Abstract

Non-thermal atmospheric gas plasma (AGP) exhibits cytotoxicity against malignant cells with minimal cytotoxicity toward normal cells. However, the mechanisms of its tumor-selective cytotoxicity remain unclear. Here we report that AGP-activated medium increases caspase-independent cell death and mitochondrial network collapse in a panel of human cancer cells, but not in non-transformed cells. AGP irradiation stimulated reactive oxygen species (ROS) generation in AGP-activated medium, and in turn the resulting stable ROS, most likely hydrogen peroxide (H_2_O_2_), activated intracellular ROS generation and mitochondrial ROS (mROS) accumulation. Culture in AGP-activated medium resulted in cell death and excessive mitochondrial fragmentation and clustering, and these responses were inhibited by ROS scavengers. AGP-activated medium also increased dynamin-related protein 1-dependent mitochondrial fission in a tumor-specific manner, and H_2_O_2_ administration showed similar effects. Moreover, the vulnerability of tumor cells to mitochondrial network collapse appeared to result from their higher sensitivity to mROS accumulation induced by AGP-activated medium or H_2_O_2_. The present findings expand our previous observations on death receptor-mediated tumor-selective cell killing and reinforce the importance of mitochondrial network remodeling as a powerful target for tumor-selective cancer treatment.

## INTRODUCTION

Non-thermal atmospheric gas plasma (AGP) has emerged as a promising alternative approach to conventional genotoxic therapies, because AGP irradiation specifically kills tumor cells while sparing normal cells under optimal conditions [1–4]. AGP irradiation preferentially activates various cell death modalities, including apoptosis, necrosis, and autophagy, in cancer cell lines and primary cancerous cells and tissues compared with their normal counterparts [5–8]. AGP irradiation also inhibits the cell proliferation, migration, and invasion of several cancer cell lines and reduced cell growth in an *in vivo* xenograft model [5, 9, 10]. AGP generated from a variety of gas types is capable of killing tumor cells. AGP has been shown to cause cell cycle arrest and DNA damage checkpoint responses and to alter gene expression profiles [10–13]. Reactive oxygen/nitrogen species (ROS/RNS) generation and/or reductions in antioxidant systems are associated with most tumor cell killing by AGP, and therefore oxidative stress is suggested to play a key role in the antitumor activity [5, 14–17].

Recently, culture with AGP-activated medium was demonstrated to be effective for killing of various tumor cells, such as glioblastoma, chemoresistant ovarian, gastric, and pancreatic cancer cells, *in vitro* and *in vivo* while exhibiting minimal cytotoxicity toward normal cells [18–21]. Indirect AGP treatment appears to share many biological activities with direct AGP irradiation, including apoptosis induction and ROS generation [20, 21]. However, compared with direct AGP irradiation, little is known about the mechanisms for the antitumor activity of indirect AGP treatment, and the molecular basis of its tumor-selectivity remains unclear.

Mitochondria are highly dynamic organelles with a reticular network that is delicately balanced between two antagonistic machineries responsible for fission and fusion of the mitochondrial membrane. The mitochondrial network is critical for cell function and apoptosis [22, 23], because a defect in either fission or fusion causes severe mitochondrial and cellular dysfunctions. Mitochondrial fission helps to eliminate damaged mitochondria through mitophagy [24], such that disruption of mitochondrial fission leads to an extensively interconnected and collapsed mitochondrial network, and defects in mitochondrial quality control. Meanwhile, mitochondrial fusion facilitates the exchange of mitochondrial DNA and metabolites required for mitochondrial function [25]. Consequently, defects in mitochondrial fusion lead to mitochondrial fragmentation and loss of mitochondrial DNA [26], reduced growth, decreased mitochondrial membrane potential (ΔΨm), and defective respiration [27]. In mammalian cells, mitochondrial fusion and fission are controlled by dynamin-related proteins with GTPase activity, namely mitofusin 1/2 (Mfn1/2), optic atrophy 1 (OPA1), and dynamin-related protein 1 (Drp1). Mfn1/2, and OPA1 act in concert to regulate mitochondrial fusion and cristae organization, while Drp1 regulates mitochondrial fission [22, 23].

We previously demonstrated that TNF-related apoptosis-inducing ligand (TRAIL), a highly tumor-selective anticancer drug, induces aberrant mitochondrial network changes in cancer cells, but not in non-transformed cells [28]. The mitochondria within tumor cells specifically undergo excessive mitochondrial fragmentation followed by clustering. This mitochondrial network collapse is paralleled by apoptosis and mitochondrial ROS (mROS) accumulation stimulated it. By analogy with TRAIL in terms of the tumor-selective cytotoxicity and involvement of ROS, we hypothesized that AGP also targets mitochondrial network remodeling for its cytotoxicity. We developed a non-thermal AGP jet, established an *in vitro* model to examine the antitumor activity of AGP-activated medium, and elucidated the mechanisms of action within the context of tumor-selectivity. Here we show that AGP-activated medium exhibits cytotoxicity toward chemoresistant cancer cells such as malignant melanoma, non-small cell lung cancer (NSCLC), and osteosarcoma cells while sparing non-transformed cells. We also demonstrate that indirect AGP treatment preferentially stimulates mitochondrial network collapse in tumor cells compared with non-transformed cells through their vulnerability to mitochondrial mROS accumulation and ROS-mediated mitochondrial network remodeling.

## RESULTS

### AGP-activated medium exhibits cytotoxicity against a panel of human cancer cell lines, but not non-transformed cells

**Figure 1:**
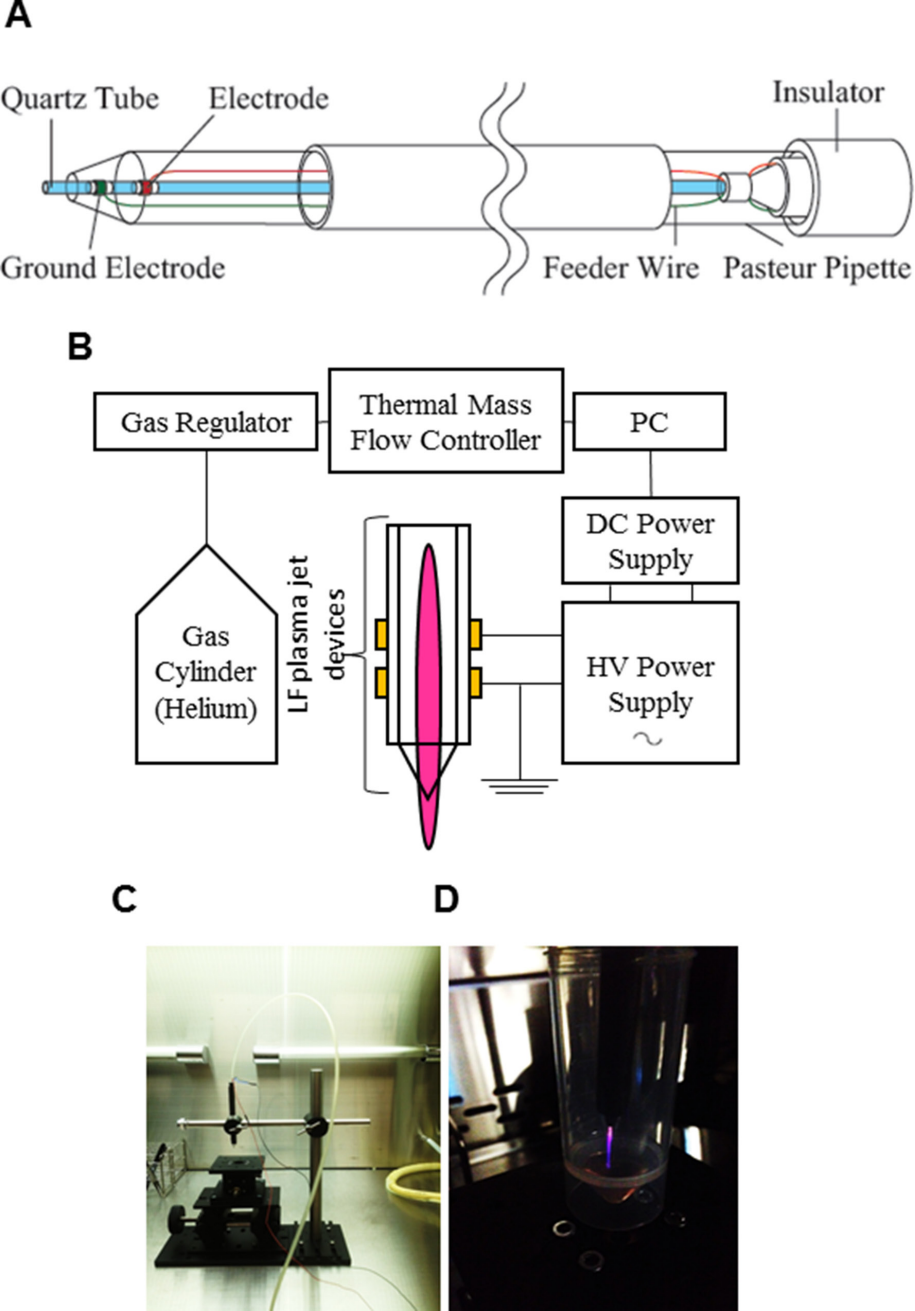
Experimental setup for AGP generation and preparation of AGP-activated medium (**A, B**) Schematic diagrams of the AGP jet device (A) and the whole AGP generation system operated in helium gas (B). (**C, D**) Photos of the AGP jet device (C) and a typical image of AGP generated by DBD of helium gas (**D**).

**Figure 2:**
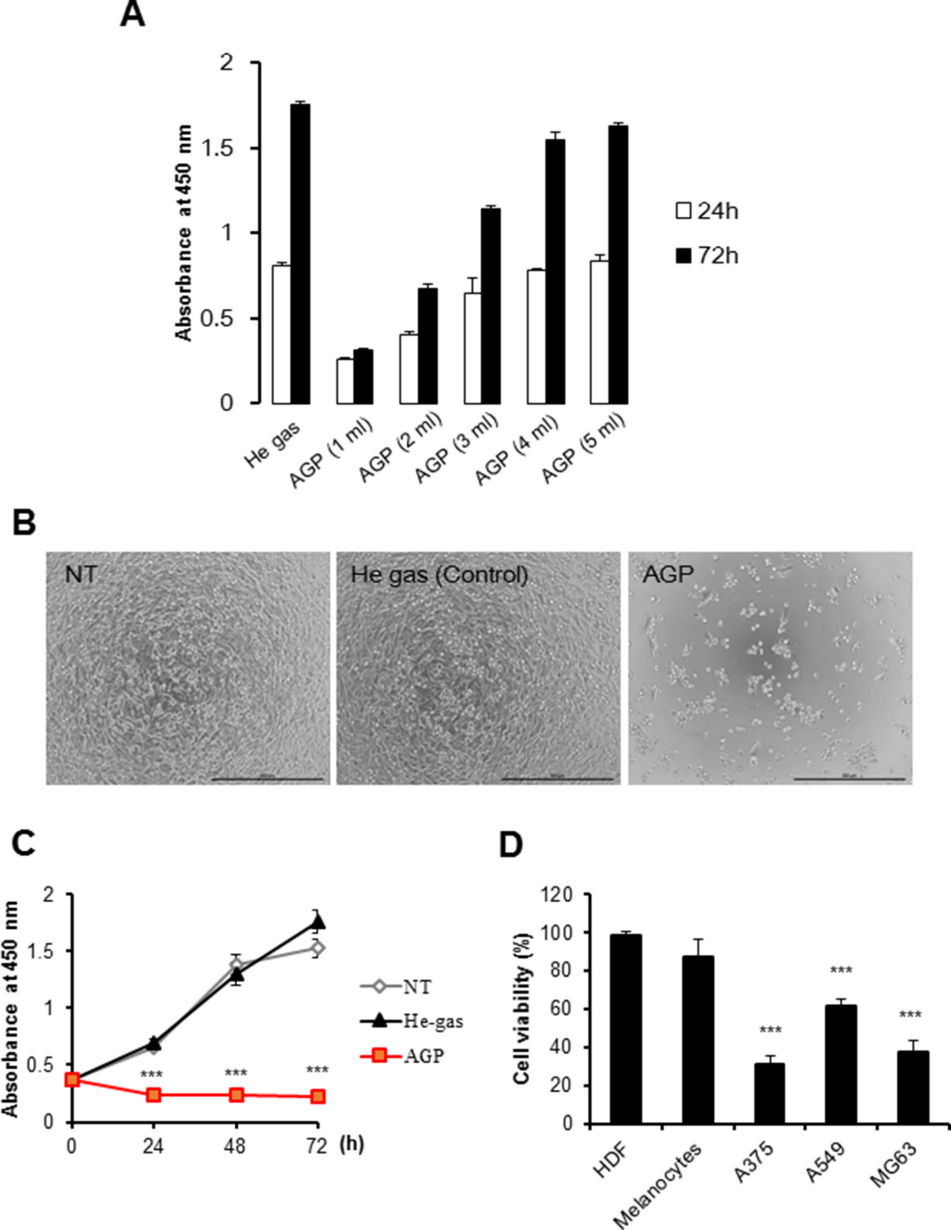
AGP-activated medium exhibits tumor-selective cytotoxicity (**A**) Different volumes of DMEM (1–5 ml) were irradiated with AGP. A375 cells were cultured in each AGP-activated medium for 24 or 72 h, and measured for their cell growth (absorbance at 450 nm) using a cell proliferation assay kit. Control medium was prepared by exposure to helium gas without discharge, and used for cell culture. The data represent means ± SEM of three independent experiments (**B**) Representative phase-contrast images of cells cultured in non-treated (NT) (left), control (middle), or AGP-activated (right) medium for 72 h. Bars = 500 μm. (**C**) A375 cells were cultured in NT, control, or AGP-activated medium for 24, 48, and 72 h, and measured for their cell growth (absorbance at 450 nm). (**D**) Cancer cells (A375, A549, MG63) and non-transformed cells (HDFs, melanocytes) were cultured in control or AGP-activated medium for 72 h, and measured for their cell viability using the WST-8 assay. The data shown are percentages of the value in control cells set at 100, and represent means ± SEM of three independent experiments. ***P < 0.001.

AGP was generated at room temperature using a low-frequency (LF) plasma jet device by discharging helium gas under atmospheric conditions (Figure 1A–1D) and used to irradiate Dulbecco's minimum essential medium (DMEM). The target cells were then cultured in the AGP-activated medium for 24 or 72 h, and assessed for their cell growth. When AGP-activated medium was prepared using different volumes of DMEM (1–5 ml), the cytotoxicity of the resulting medium against human malignant melanoma A375 cells decreased as the volume increased (Figure 2A). AGP-activated medium prepared with DMEM at volumes of ≥ 4 ml exhibited no substantial cytotoxicity at 72 h. Meanwhile, AGP irradiation to 1 ml of DMEM for 5 min, but not 1 min, reproducibly produced highly toxic medium, which almost completely (maximum of 90%) decreased the cell viability at 24 h. Therefore, we applied this protocol throughout the present study. Culture in AGP-activated medium for 24 h considerably increased morphologically damaged and detached cells (Figure 2B). In contrast, control medium exposed to helium gas without discharge exhibited minimal cytotoxicity. Consistent with the microscopic observations, AGP-activated medium significantly decreased the cell viability during the initial 24 h and this decrease further developed over time up to 72 h (Figure 2C). AGP-activated medium also exhibited significant cytotoxicity against all human malignant tumor cells examined, including A549 (NSCLC) and MG63 (osteosarcoma) cells, but showed little cytotoxicity against non-transformed cells such as human dermal fibroblasts (HDFs) and melanocytes (Figure 2D).

### AGP-activated medium increases caspase-independent cell death in a tumor-specific manner

**Figure 3:**
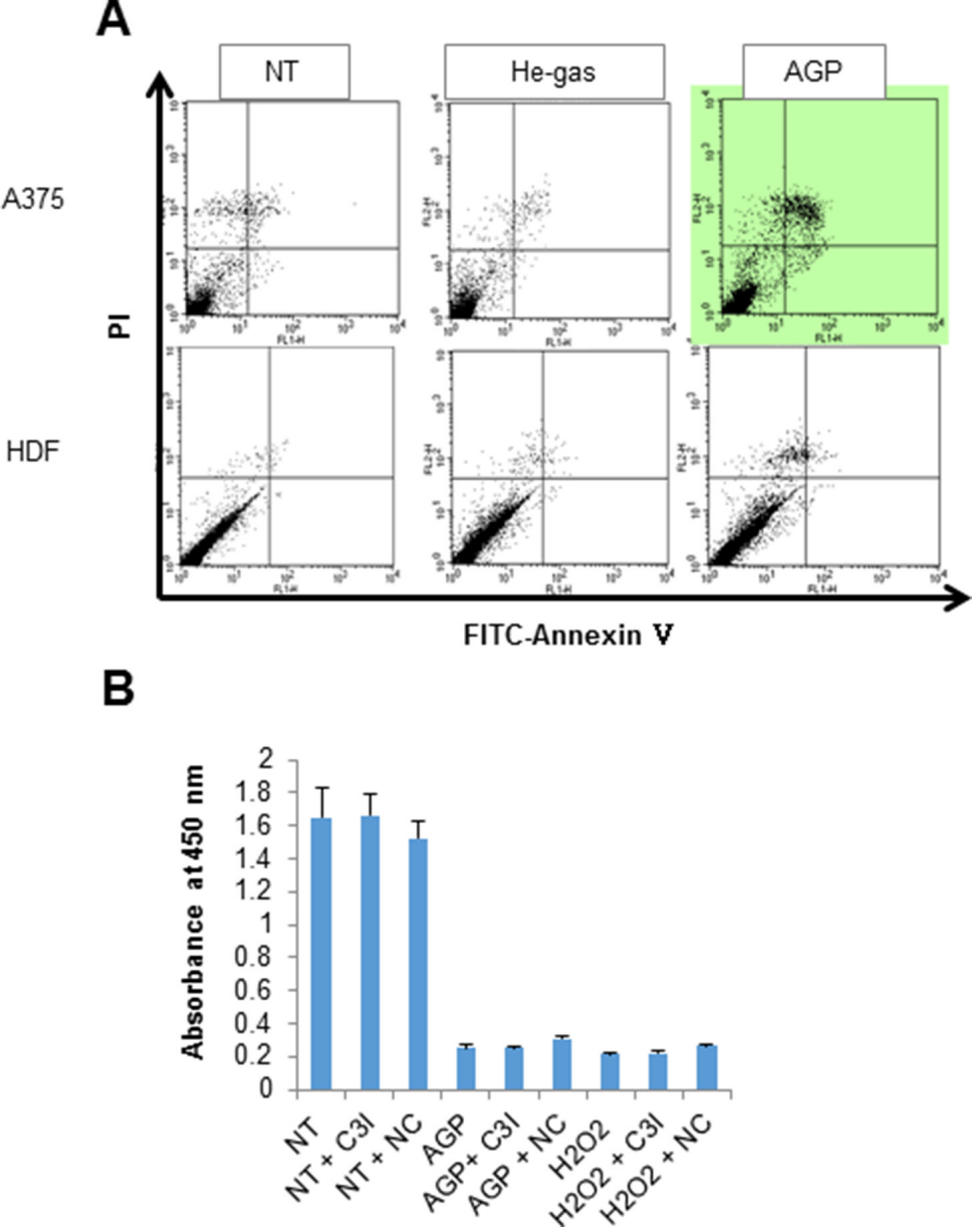
AGP-activated medium induces caspase-independent cell death (**A**) A375 cells and HDFs were cultured in control or AGP-activated medium for 48 h, stained with annexin V-FITC and PI, and analyzed by flow cytometry. Representative histograms of three independent experiments with similar results are shown. (**B**) A375 cells were treated with non-treated medium (NT), AGP-activated medium (AGP), or H_2_O_2_ (100 μM) for 72 h in the absence or presence of the caspase-3 inhibitor z-DEVD-FMK (10 μM) or necrostatin-1 (100 μM), and measured for their cell viability in triplicates. The data represent means ± SEM of a representative of three independent experiments with similar results.

Next, we attempted to determine the cell death modalities induced by AGP-activated medium. Staining with fluorescein isothiocyanate (FITC)-conjugated annexin V and propidium iodide (PI) followed by flow cytometric analysis revealed that AGP-activated medium considerably increased annexin V-positive cells in A375 cells, but not in HDFs (Figure 3A). However, the cell death induced by AGP-activated medium was not inhibited by treatment with the caspase-3/7-specific inhibitor z-DEVD-fluoromethylketone (FMK) (Figure 3C) or general caspase inhibitor z-VAD-FMK up to 100 μM for 24 h (data not shown). These results indicate that AGP-activated medium increases caspase-independent cell death in a tumor-specific manner. As reported previously [29], treatment with 100 μM H_2_O_2_ resulted in considerable cell death, which was quite resistant to the caspase inhibitors (Figure 3C). In addition, necrostatin up to 100 μM had minimal effects on the cell death triggered by AGP-activated medium or H_2_O_2_.

### Role of cell-independent and cell-dependent ROS generation in AGP cytotoxicity

**Figure 4:**
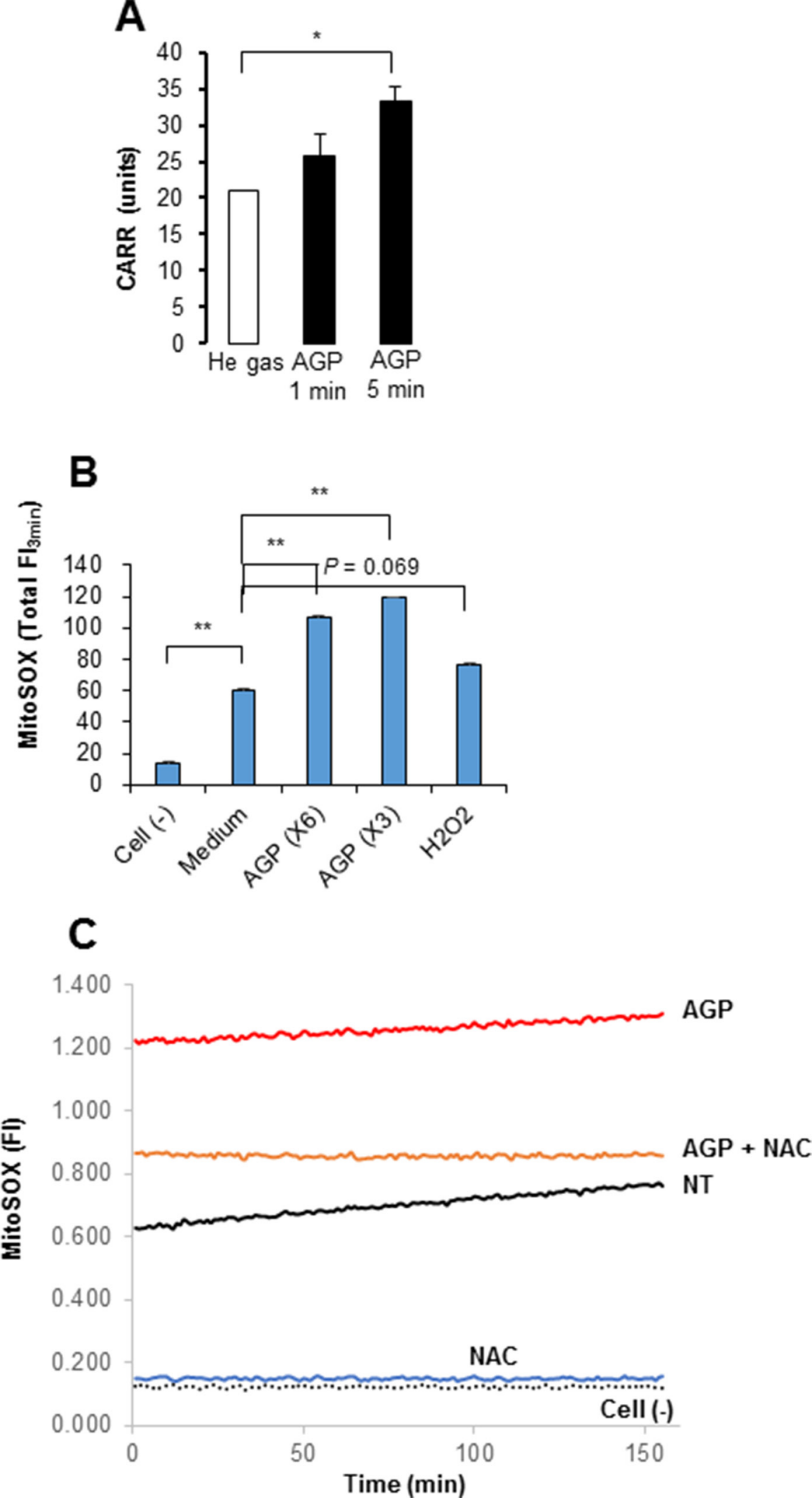
Generation of ROS by AGP irradiation and AGP-activated medium (**A**) DMEM (1 ml) was exposed to AGP or helium gas without discharge for 1 or 5 min, and measured for its free radical levels by the d-ROMs test using an active oxygen and free radicals automatic analyzer. In this analysis, 1 CARR unit was corresponded to 0.08 mg/dl H_2_O_2_. The data represent means ± SEM of three different experiments. *P < 0.05. (**B**) A375 cells suspended in HBSS were incubated with 3- or 6-fold diluted AGP-activated medium or H_2_O_2_ (100 μM) for 4 h at 37°C, and loaded with MitoSOX^™^ Red. The cells were washed, resuspended in HBSS and then fluorescence was measured at 5– s intervals for up to 3 min in a microplate fluorescence reader with excitation and emission at 542 and 592 nm, respectively. The data were expressed as the total FI for 3 min and represent means ± SEM of three different experiments. **P < 0.01. (**C**) A375 cells suspended in HBSS were incubated with AGP-activated medium in the absence or presence of NAC (2 mM), and measured for their MitoSOX^™^ Red signal for 3 min as described above. The data are representative of three independent experiments with similar results.

**Figure 5:**
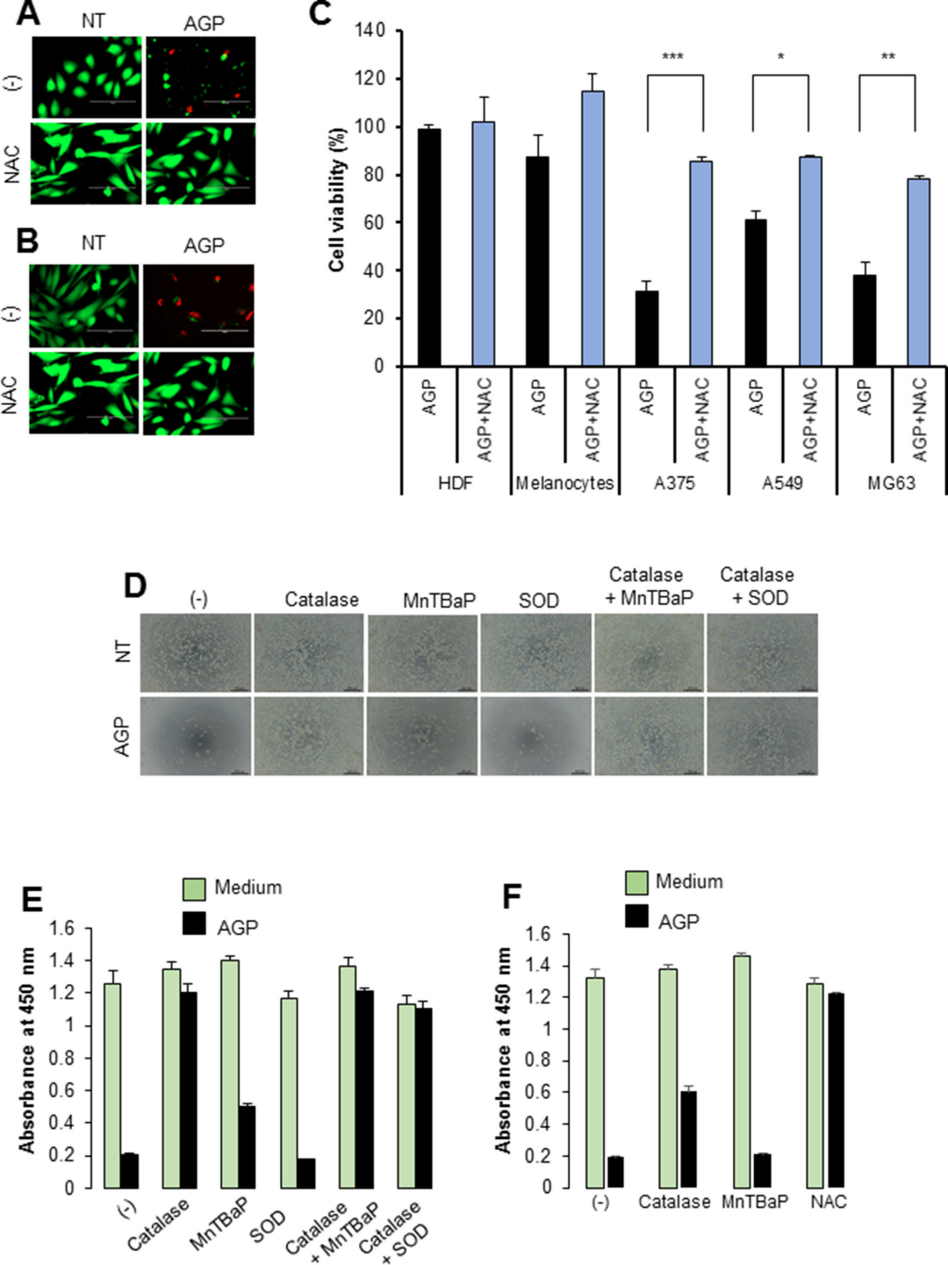
Role of ROS in the cell death induced by AGP-activated medium (**A, B**) A375 cells (A) and A2058 cells (B) were placed in an 8-well chambered coverglass and incubated with AGP-activated medium in the absence or presence of NAC (2 mM) for 24 h at 37°C in a 5% CO_2_ incubator. The cells were then stained with 4 μM each of calcein-AM and EthD-1 and observed under an EVOS FL Cell Imaging System equipped with a digital inverted microscope at × 300 magnification. (**C**) Cancer cells (A375, A549, MG63) and non-transformed cells (HDFs, melanocytes) were cultured in AGP-activated medium in the absence or presence of NAC (2 mM) for 72 h, and measured for their cell viability using the WST-8 assay. The data shown are percentages of the value in control cells set at 100 and represent means ± SEM of three independent experiments. *P < 0.05; **P < 0.01; ***P < 0.001. (**D**) Representative phase-contrast images of A375 cells cultured in non-treated (NT) and AGP-activated medium in the absence or presence of catalase (10 U/ml), SOD (10 U/ml), and MnTBaP (30 μM) alone or in combination for 48 h. (**E, F**) The viabilities of A375 cells (E) and A2058 cells (F) were measured under the same experimental conditions as in (D). The data represent means ± SEM of four independent experiments.

Since ROS are suggested to be responsible for AGP cytotoxicity against cancer cells [5, 14–17], we examined the role of ROS in the cytotoxicity of AGP-activated medium. First, we measured the free radical levels in AGP-activated medium using the derivatives of reactive oxygen metabolite (dROM) test in an automatic active oxygen and free radical analyzer. The results showed that the levels of ROS/free radicals were significantly elevated in AGP-activated medium prepared by 5 min, but not 1 min, of AGP irradiation [levels for control (helium gas-treated), 1 min, and 5 min media were 21.5 ± 0.7, 25.7 ± 3.2, and 33.3 ± 1.9 CARR units, respectively (N = 3), where 1 CARR unit corresponded to 0.08 mg/dl (240 μM) H_2_O_2_) (Figure 4A), indicating that AGP irradiation directly produces ROS in the medium in a cell-free manner. Next, we examined whether AGP-activated medium stimulated intracellular ROS levels. We focused on mROS accumulation, because mitochondrial oxidative stress is a major cause of mitochondrial integrity collapse. The mROS accumulation was measured in a microplate fluorescence reader using the highly mitochondria-targeted fluorescent probe MitoSOX Red. AGP-activated medium and, to a lesser extent, 100 μM H_2_O_2_ increased the mROS levels in A375 cells compared with non-treated (NT) control medium (Figure 4B). The effect of AGP-activated medium was observed in a dose-dependent manner and completely abolished by treatment with 2 mM *N*-acetylcysteine (NAC) (Figure 4C). Substantial MitoSOX Red signals were observed in NT medium-treated cells compared with cell-free controls, which were reduced to the level of the cell-free sample by NAC, indicating ambient mROS accumulation in the cells. Essentially the same results were obtained for A2058, SAOS-2, and HOS cells (data not shown). Taken together, these results indicate that AGP-activated medium induces mROS accumulation in a cell-dependent manner. Next, we determined the role of ROS in the cytotoxicity of AGP-activated medium. First, we assessed the effect of ROS scavengers on the cytotoxicity. Dead and damaged cells with compromised cell membranes were stained red with ethidium bromide homodimer-1 (EthD-1), while live cells were stained green with calcein-acetoxymethyl ester (AM). AGP-activated medium markedly decreased green fluorescence and substantially increased red fluorescence in A375 and A2058 cells, and these effects were completely abolished by the presence of NAC (Figure 5A and 5B). Cell viability measurements validated the microscopic observations, and expanded the protective effect of NAC to other tumor cell types such as A549 and MG63 cells (Figure 5C). The slight reduction in the cell viability of melanocytes was also recovered by NAC treatment, suggesting a common role of ROS in tumor and non-transformed cells. To characterize the oxidant species involved, we examined the effects of different species-specific ROS scavengers on the cytotoxicity. The H_2_O_2_-scavenging enzyme catalase almost completely blocked the cell damage and reduction in cell viability induced by AGP-activated medium. Meanwhile, MnTBaP, a cell-permeable scavenger of superoxide-derived oxidants, partially inhibited these effects, and the cell-impermeable enzyme SOD was ineffective (Figure 5D and 5E). No additional inhibitory effect was observed when catalase and MnTBaP were applied together. However, NAC and catalase, but not MnTBaP, inhibited the reduction in the viability of A2058 cells (Figure 5F). These results indicate that both cell-dependent and cell-independent ROS generation pathways are involved in the tumor-selective cytotoxicity of AGP-activated medium.

### AGP-activated medium induces mitochondrial network collapse in a tumor-specific manner

**Figure 6:**
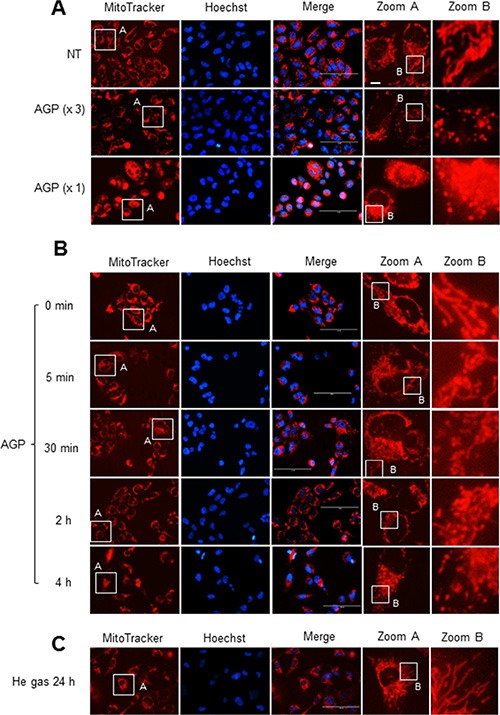
AGP-activated medium induces mitochondrial network collapse in cancer cells A375 cells in FBS/DMEM were plated in an 8-well chambered coverglass and treated with undiluted or 3-fold diluted AGP-activated medium for 24 h (**A**) or undiluted AGP-activated medium for the indicated times (**B**) or control medium for 24 h (**C**) at 37°C. After washing, the cells were stained with MitoTracker Red CMXRos for 1 h, and washed again. Images were obtained and analyzed using an EVOS FL Cell Imaging System at × 1200 magnification.

**Figure 7:**
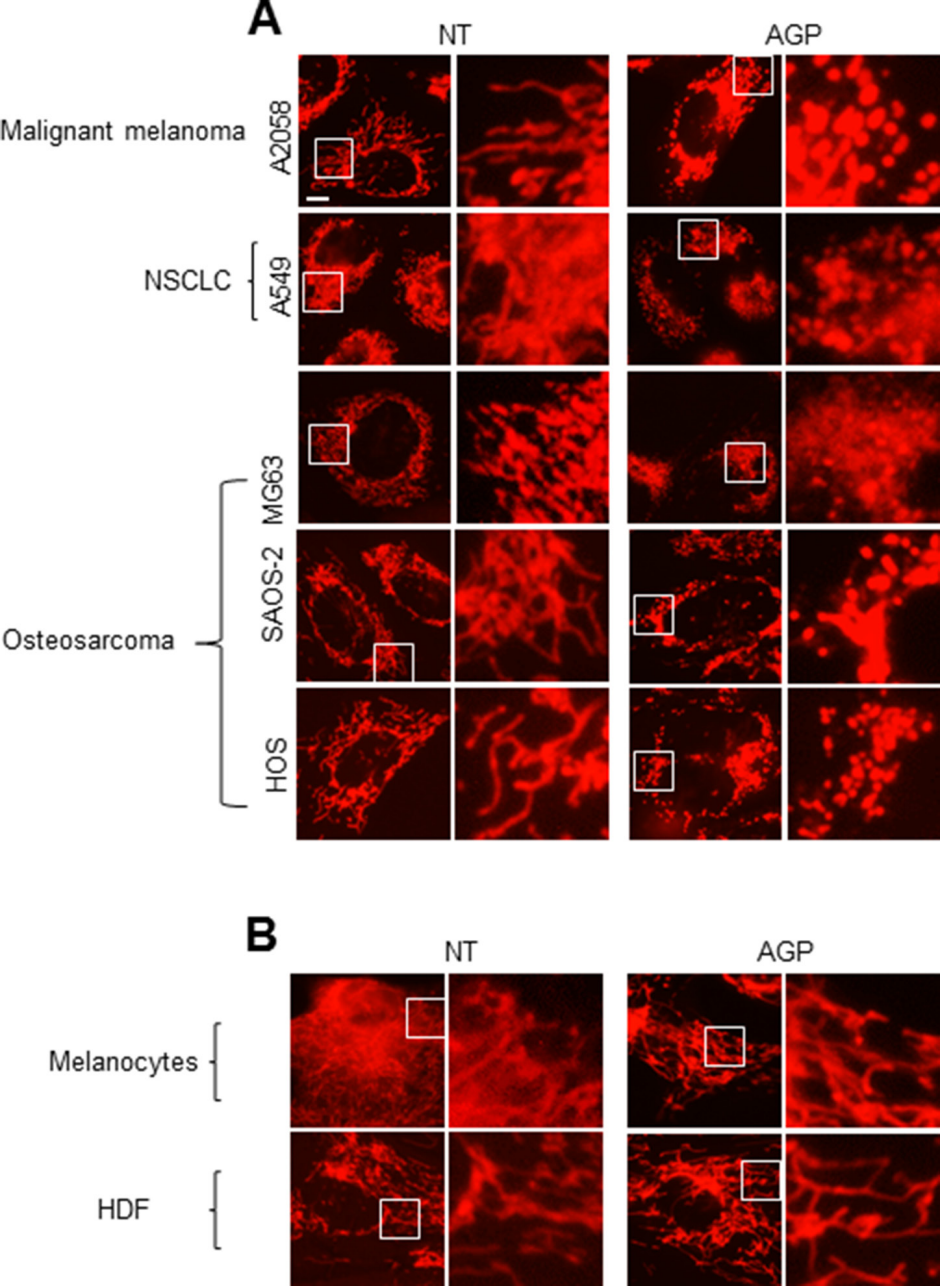
The mitochondrial network collapse is tumor-specific (**A, B**) A2058, A549, MG63, SAOS-2, and HOS cells (A) and melanocytes and HDFs (B) were treated with AGP-activated medium for 24 h at 37°C, stained with MitoTracker Red CMXRos for 1 h, and analyzed for their mitochondrial network. × 1200 magnification.

To determine the possible effect of AGP-activated medium on the mitochondrial network in cancer cells, cells were stained with the mitochondria-targeting dye MitoTracker Red CMXRos, and then mitochondrial network morphology was analyzed under a fluorescence microscope. In control A375 cells, most mitochondria showed a reticular network ranging from the nucleus, a feature of well-balanced mitochondrial fission and fusion (Figure 6A, top). AGP-activated medium exhibited different effects depending on the time of treatment and concentration. At 24 h, low concentrations (3– 6-fold dilution) of AGP-activated medium resulted in an excessive mitochondrial fragmentation, thereby producing small round mitochondria, but caused minimal clustering (Figure 6A, middle). Meanwhile, higher concentrations of AGP-activated medium caused additional heavy clustering of the fragmented mitochondria (Figure 6A, bottom). The time course experiments revealed that in the initial phase (5 min to 2 h), only modest mitochondrial fragmentation was observed in a small number of mitochondria. Subsequently, a higher extent of mitochondrial fragmentation became pronounced over time. As a result, most mitochondria lost their reticular network and became punctate and clustered at 4– 24 h (Figure 6B). In contrast, control medium caused minimal mitochondrial fragmentation and clustering up to 24 h (Figure 6C). Similar mitochondrial network collapse was observed in A2058, A549, MG63, SAOS-2, and HOS cells, but not in melanocytes and HDFs (Figure 7A and 7B). These results indicate that AGP-activated medium induces mitochondrial network collapse in a tumor-specific manner.

### AGP-activated medium induces Drp1-mediated mitochondrial network remodeling in a tumor-specific manner

**Figure 8:**
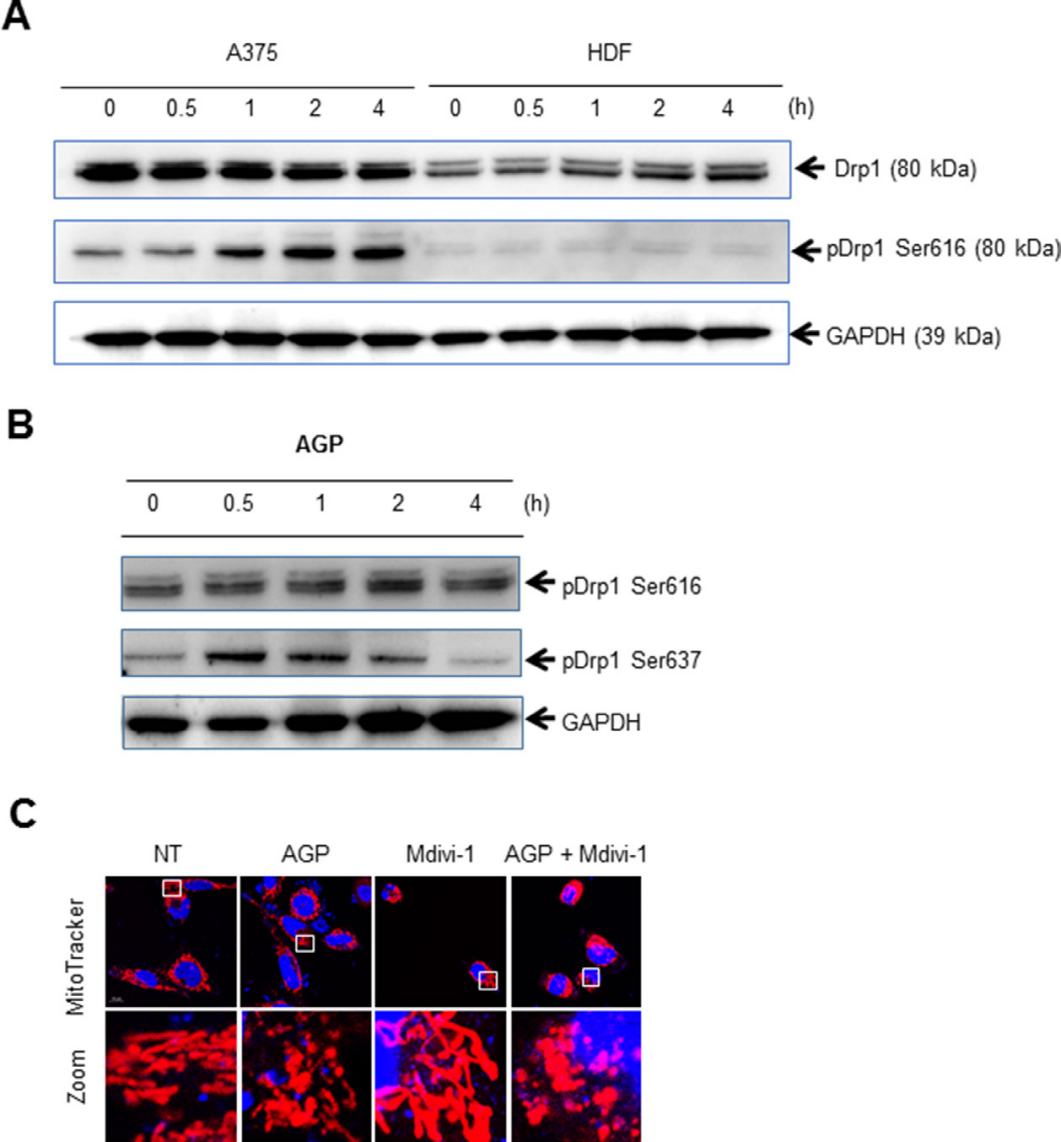
AGP-activated medium induces Drp1-mediated mitochondrial remodeling (**A, B**) A375 cells and HDFs (A, B) or A375 cells (B) were incubated with AGP-activated medium for the indicated times, and analyzed for their expression of pDrp1 Ser616 (A) and pDrp1Ser637 (B) by immunoblotting. GAPDH was evaluated as a loading control. (**C**) A375 cells were treated with AGP-activated medium in the absence or presence of mdivi-1 (50 μM) for 24 h at 37°C, and analyzed for their mitochondrial network morphology. Mitochondria and nuclei were stained with MitoTracker Red and Hoechst 33342, respectively, and observed under a confocal microscope.

Drp1 plays a key role in the control of mitochondrial network dynamics, because its phosphorylation at Ser616 and Ser637 positively and negatively regulates mitochondrial fission, respectively [22, 23]. Immunoblotting analyses with an antibody against Drp1 phosphorylated at Ser616 (pDrp1 Ser616) revealed that AGP-activated medium substantially increased the level of pDrp1 Ser616 in A375 cells. This effect became pronounced at 1 h, and then further developed over time until at least 4 h (Figure 8A, top). A lower level of pDrp1 Ser616 was observed in unstimulated HDFs, and this level was unaffected by AGP-activated medium (Figure 8A, middle). Meanwhile, AGP-activated medium increased the level of pDrp1 Ser637 in A375 cells as rapidly as 30 min, with the level reaching its maximum at 30 min to 1 h and thereafter declining to the basal level (Figure 8B). However, minimal changes in the levels of pDrp1 Ser637 were observed in HDFs (data not shown). These results indicate that AGP-activated medium induces Drp1-mediated mitochondrial network remodeling in a tumor-specific manner. To determine the role of the mitochondrial network remodeling in the mitochondrial network collapse, we examined the effects of the Drp1 inhibitor mdivi-1, which blocks mitochondrial fission in cancer cells [28, 30]. As shown in Figure 8C, treatment of A375 cells with mdivi-1 resulted in elongated, highly-interconnected mitochondria, a hallmark of mitochondrial fission inhibition. Nevertheless, the mitochondrial network collapse was not blocked by mdivi-1 treatment.

### H_2_O_2_ mediates the mitochondrial network collapse and tumor-selective cytotoxicity

**Figure 9:**
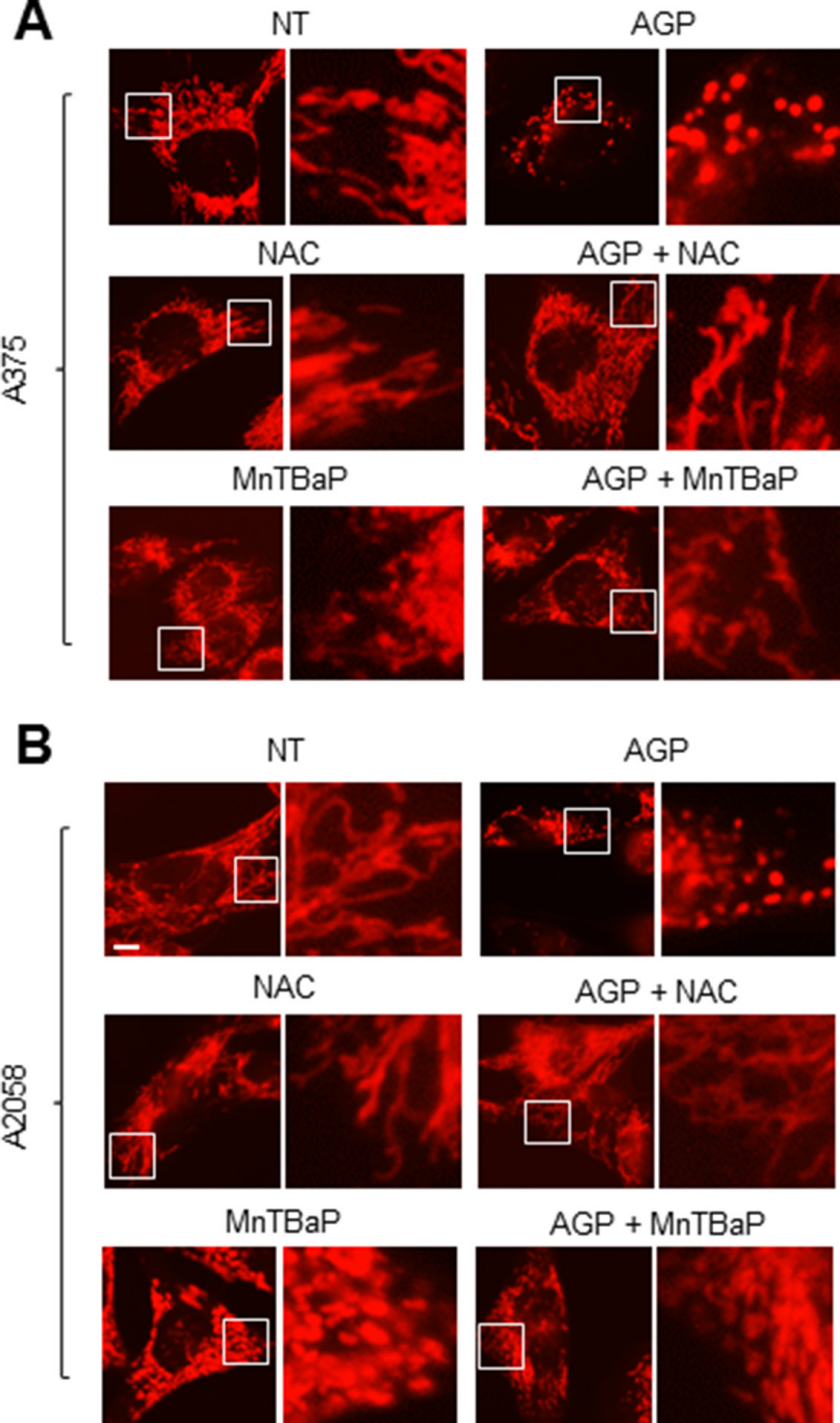
Role of ROS in the mitochondrial network collapse (**A, B**) A375 cells (A) and A2058 cells (B) were incubated with AGP-activated medium in the absence or presence of NAC (2 mM) or MnTBaP (30 μM) for 24 h at 37°C in a 5% CO_2_ incubator, and analyzed for their mitochondrial network morphology. × 1200 magnification.

Next, we examined the role of ROS in the mitochondrial network by analyzing the effects of ROS scavengers. Treatment with NAC completely blocked the mitochondrial network collapse in A375 cells, while MnTBaP inhibited the collapse to a lesser extent (Figure 9A). Furthermore, NAC completely inhibited the mitochondrial network collapse, while MnTBaP had a minimal effect in A2058 cells (Figure 9B). Unlike NAC, MnTBaP alone moderately increased the amounts of truncated and highly-interconnected mitochondria in both cell types. These results indicate that ROS mediate the mitochondrial network collapse.

**Figure 10:**
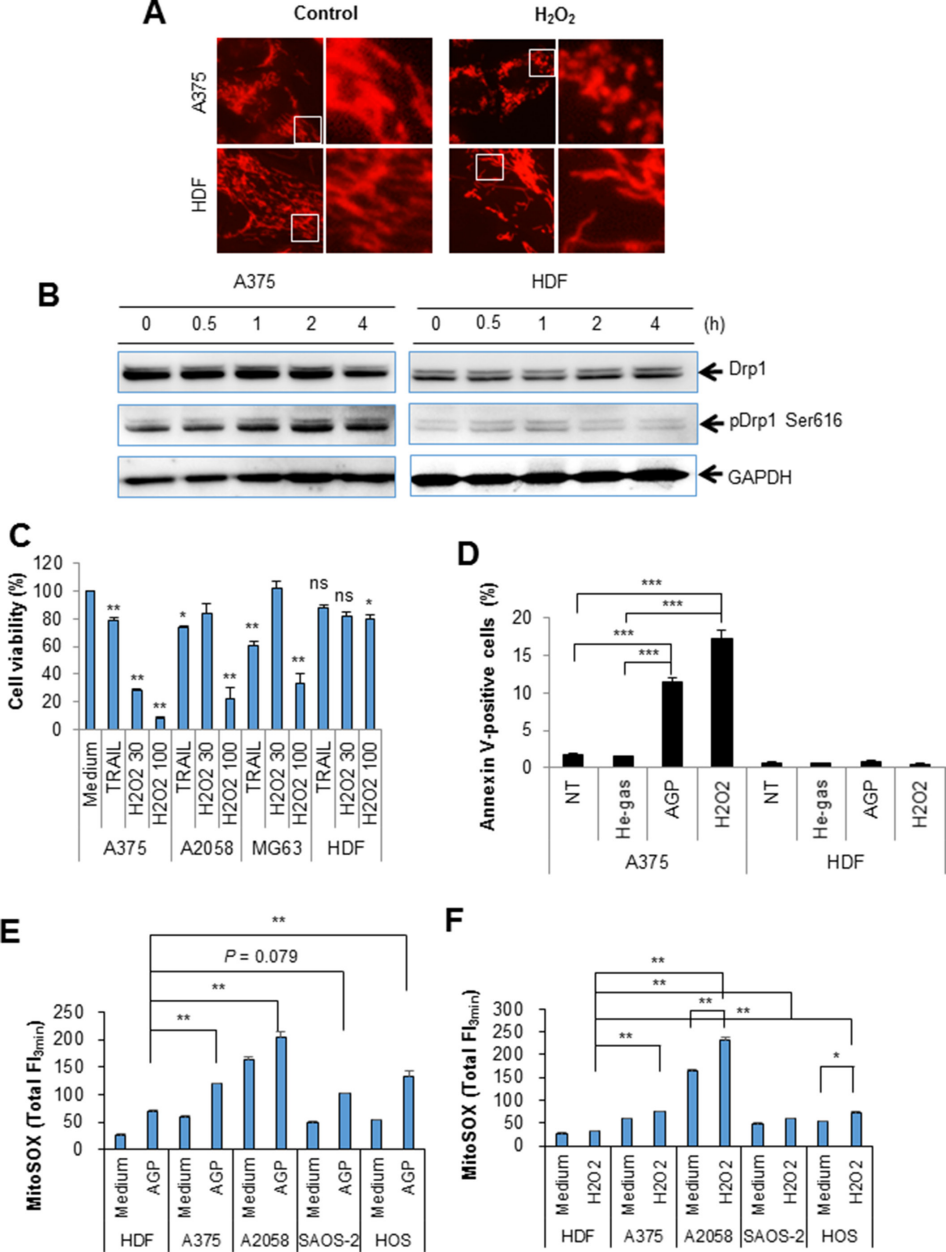
H_2_O_2_ mimics the tumor-specific mitochondrial network collapse, increased pDrp1 Ser616, cytotoxicity, and mROS accumulation (**A**) A375 cells were incubated with H_2_O_2_ (100 μM) for 24 h at 37°C in a 5% CO_2_ incubator, and analyzed for their mitochondrial network morphology. × 1200 magnification. (**B**) A375 cells and HDFs were incubated with H_2_O_2_ (100 μM) for the indicated times, and analyzed for their expression of pDrp1 Ser616 by immunoblotting. GAPDH was evaluated as a loading control. (**C**) A375, A2058, and MG63 cells, and HDFs were treated with H_2_O_2_ (30 or 100 μM) or TRAIL (100 ng/ml) for 24 h at 37°C in a 5% CO_2_ incubator, and measured for their cell viability was by the WST-8 assay. The data shown are percentages of the value in control cells set at 100 and represent means ± SEM of three independent experiments. *P < 0.05; **P < 0.01; ns, not significant. (**D**) A375 cells and HDFs were treated with non-treated (NT) control, or AGP-activated medium or H_2_O_2_ (100 μM) for 24 h at 37°C in a 5% CO_2_ incubator, stained with annexin V-FITC/PI, and analyzed by flow cytometry. The data represent the means ± SE of three independent experiments. **P < 0.01; ***P < 0.001. (**E, F**) A375, A2058, SAOS-2, and HOS cells, and HDFs were treated with AGP-activated medium (E) or H_2_O_2_ (100 μM) (F) for 4 h at 37°C in a 5% CO_2_ incubator, and measured for their MitoSOX^™^ Red signals for 3 min. The data represent means ± SEM of three different experiments. *P < 0.05; **P < 0.01.

Since the results of the dROM test suggested that H_2_O_2_ generation occurred in AGP-activated medium, we examined its roles in the mitochondrial network collapse and tumor-specific cytotoxicity. Treatment with 100 μM H_2_O_2_ for 24 h resulted in heavy mitochondrial fragmentation and clustering in A375 cells, but caused only modest mitochondrial fragmentation in HDFs (Figure 10A). Moreover, H_2_O_2_ considerably increased the level of pDrp1 Ser616 in A375 cells, but not in HDF (Figure 10B). In addition, H_2_O_2_ dose-dependently reduced cell viability in various tumor cell types such as A375, A2058, and MG63 cells, while it had only a modest cytotoxicity in HDFs (Figure 10C). In agreement with these observations, annexin V-positive cells were significantly increased in H_2_O_2_-treated A375 cells, but not in HDFs (Figure 10D). Collectively, these results indicate that ROS mainly H_2_O_2_ mediate the mitochondrial network collapse and tumor-selective cytotoxicity. Our previous study demonstrated that tumor cells are more prone to TRAIL-triggered mROS accumulation than normal cells [28]. Therefore, we compared the effect of AGP treatment on mROS accumulation between the two cell types. MitoSOX measurements revealed that tumor cells with different origins commonly displayed significantly higher levels of mROS accumulation in the resting state, and higher levels of mROS accumulation were observed after AGP treatment and H_2_O_2_ exposure in various tumor cell types compared with HDFs (Figure 10E and 10F). These results indicate that H_2_O_2_ mediates the tumor-specific mitochondrial network collapse and cytotoxicity.

## DISCUSSION

The present study demonstrated that AGP-activated medium is highly cytotoxic against chemoresistant tumor cells such as malignant melanoma, NSCLC, and osteosarcoma cells with high tumor-selectivity (Figure 2). AGP-activated medium stored at 4°C retained the cytotoxicity for a maximum of 5 days under optimal conditions. Thus, indirect AGP treatment can serve as an alternative and advantageous approach over direct AGP irradiation. The primary application of direct AGP irradiation may be limited to surface cancerous tissues because of its short outreach [4], while AGP-activated medium can be readily administered systematically or locally to tumors in deep tissues. Indirect AGP treatment is also easier to perform than direct AGP irradiation, because this method does not require an AGP jet device once AGR-activated medium is prepared. Indirect AGP treatment can also provide some useful clues for the underlying mechanisms of AGP cytotoxicity. The effectiveness of AGP-activated medium can exclude many possible causes such as heat shock, mechanical stress on the surface, and complicated physicochemical reactions among AGP and the air or fluid *in situ*. It can also eliminate the involvement of short-lived ions and radicals in the cytotoxicity. The cell death induced by AGP-activated medium occurred in a caspase-independent manner (Figure 3), thereby being different from caspase-dependent canonical apoptosis. This is similar to the cytotoxicity of another AGP-activated medium against A549 cells [21]. The resistance of the cell death to necrostatin suggest a minor role of necroptosis. Considering the canonical apoptosis induction by TRAIL, the induction of another non-canonical cell death modality by AGP may contribute to its capability for killing TRAIL-resistant cancer cell types (Figure 10), although further studies are necessary to define the cell death modalities.

**Figure 11:**
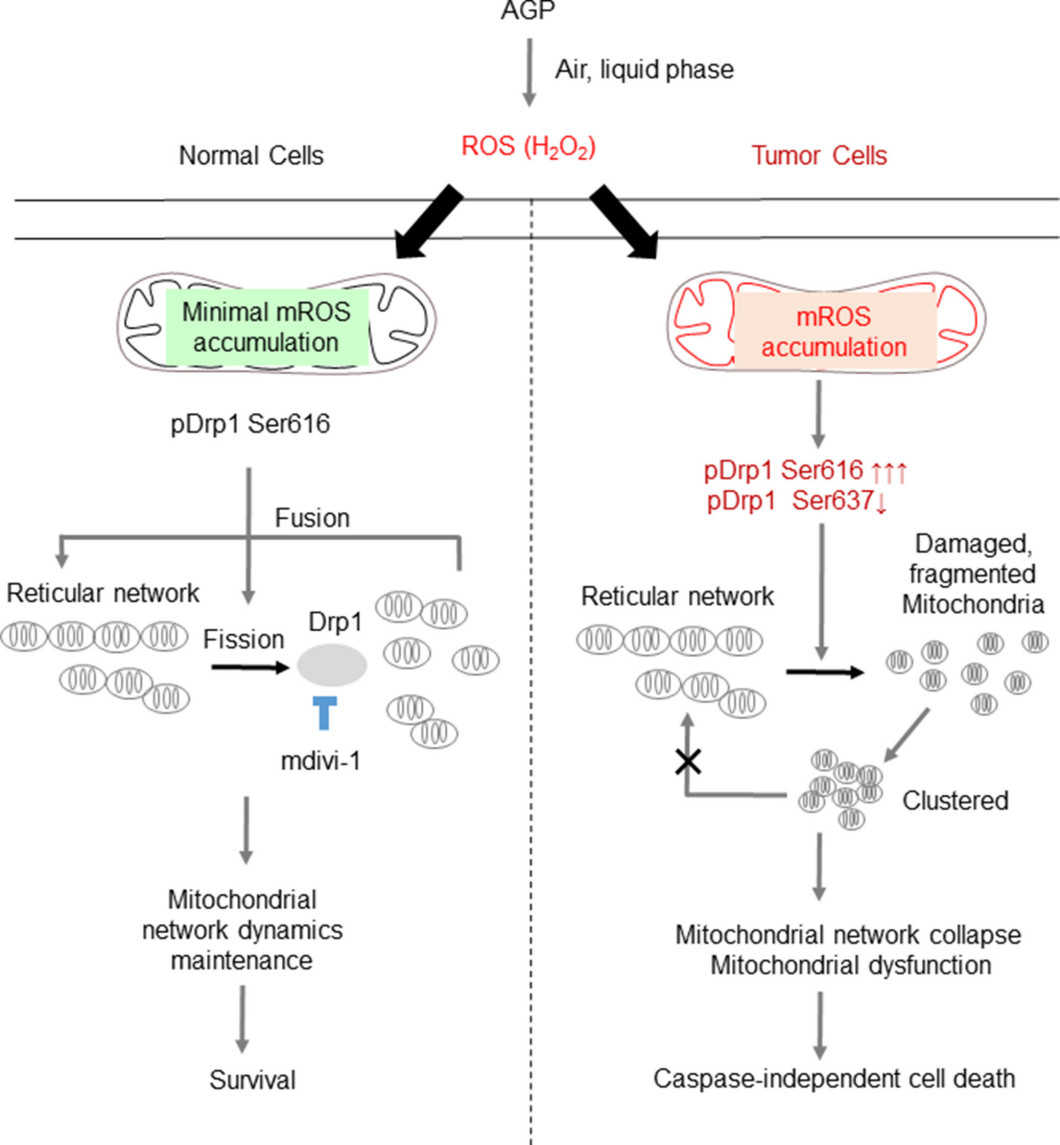
Hypothetical model for the tumor-selective mitochondrial network collapse induced by AGP-activated medium AGP irradiation induces the generation of short-lived free radicals through physicochemical interactions with air and/or the liquid phase, which results in the formation of more stable ROS (most likely H_2_O_2_) in AGP-activated medium. Owing to an imbalance in the prooxidant and antioxidant systems, tumor cells (right) are more prone than normal cells (left) to the oxidative stress caused by exogenous ROS. In turn, the oxidative stress causes excessive mitochondrial fragmentation through the predominance of the positive signal (pDrp1 Ser616) over the negative signal (pDrp1 Ser637) for the mitochondrial fission. The oxidative stress also causes substantial mROS accumulation, which may facilitate the mitochondrial network collapse and mitochondrial integrity collapse by utilizing similar mechanisms to those observed with TRAIL [28], thereby leading to cell death. Meanwhile, in normal cells, minimal mROS accumulation occurs in response to AGP-activated medium and the mitochondrial network remodeling is maintained, thereby leading to cell survival. The vulnerability of tumor cells to oxidative stress compared with normal cells can contribute to the tumor-selective killing by AGP.

Our AGP irradiation generated substantial levels of oxidants in AGP-activated medium in a cell-independent manner as reported previously for another direct or indirect AGP treatment [5, 14–17, 20, 21]. Meanwhile the medium treatment resulted in ROS generation within the target cells (Figure 4). Analyses using the mitochondria-targeting probe MitoSOX Red revealed that AGP-activated medium substantially increased ROS accumulation within the mitochondria. Since MitoSOX Red primarily reacts with superoxide, it may be the main ROS accumulated in the mitochondria in response to both AGP-activated medium and H_2_O_2_. Collectively, our data suggest that ROS are generated by two different processes. First, multiple short-lived free radicals may be produced in a cell-independent manner in AGP-activated medium through a physicochemical process, resulting in more long-lived ROS most likely H_2_O_2_. The stable ROS in turn induce mROS accumulation in a cell-dependent process (Figure 11). The general antioxidant NAC almost completely blocked the cell damage and cell death induced by AGP-activated medium in different cell types, indicating that ROS/RNS are responsible for the cytotoxicity. Meanwhile, the species-specific anti-oxidants catalase and MnTBaP reduced the cytotoxicity to different extents depending on the cell type (Figure 5), suggesting the involvement of cell type-specific prooxidant systems.

Strikingly, indirect AGP treatment dose- and time-dependently caused mitochondrial fragmentation and clustering in a tumor-specific manner (Figures 6 and 7), as observed for TRAIL treatment [28]. Strikingly, this effect was associated with tumor-specific induction of Drp1 phosphorylation at Ser616 (Figure 8), a process required for mitochondrial fission [22, 23]. Moreover, the mitochondrial network collapse was blocked by antioxidants such as NAC and MnTBaP in parallel with cell death (Figure 9). These findings suggest that the mitochondrial network collapse plays an important role in the tumor-selective cytotoxicity of indirect AGP treatment. Analyses using H_2_O_2_ offered further evidence to support this view, because like indirect AGP treatment, H_2_O_2_ killed melanoma and osteosarcoma cell lines while sparing non-transformed cells (Figure 10) in expansion of our previous observations for melanoma cells [29]. Furthermore, the tumor-selective action of H_2_O_2_ may be attributed to the tumor-specific mROS accumulation, because H_2_O_2_ can stimulate mROS accumulation in melanoma cells, but not in melanocytes [29]. In addition, the oxidant also induced mitochondrial network collapse and Drp1 phosphorylation at Ser616, in a tumor-specific manner (Figure 10). Similar to TRAIL, indirect AGP treatment caused activation of positive and negative signals for mitochondrial fission with different kinetics. Since the positive signal of Drp1 phosphorylation at Ser616 was more persistent than the negative signal of Drp1 phosphorylation at Ser637, the mitochondrial network dynamics may be biased toward excess mitochondrial fission over time, thereby resulting in mitochondrial fragmentation. However, likewise TRAIL [28], the Drp1 inhibitor mdivi-1 augmented the mitochondrial network collapse (Figure 8), suggesting that the Drp1-dependent fission process eventually counteracts the collapse. These findings are consistent with our view that the Drp1-dependent mitochondrial fission is an adaptive response of tumor cells against death-inducing stresses such as TRAIL [28]. Collectively, mitochondrial fission appears to have dual roles in the mitochondrial network collapse. This may be in accordance with the reported dual functions of mitochondrial fission in cancer cell apoptosis, i.e., it can act as both pro-apoptotic and anti-apoptotic signals, depending on the cell type and apoptotic stimuli applied [31–37]. At present, there is no model that can depict these dual functions. However, the magnitude and more likely the different consequences of the fission might contribute to the dual functions. In this context, it may be noteworthy that mitochondrial fragmentation *per se* has a minor role in cell death induction by TRAIL but mitochondrial clustering is critical [28].

H_2_O_2_ is the most likely candidate for the initial oxidant produced by AGP irradiation. This view is consistent with the report by Adachi et al. [21], who detected H_2_O_2_ in their AGP-activated medium. The amount of H_2_O_2_ produced in AGP-activated medium seemed to be comparable to or higher than the minimal concentration of H_2_O_2_ required for mROS accumulation (Figure 4). Therefore, such high levels of H_2_O_2_ may stimulate secondary ROS/RNS generation and mROS accumulation. Given that the H_2_O_2_—mROS pathway is critical for the mitochondrial network collapse and cell death, tumor cells can be expected to more prone to these oxidative events than non-transformed cells. Indeed, significantly higher levels of mROS accumulation were observed in different cancer cell types compared with non-transformed cells in response to both H_2_O_2_ and the indirect AGP treatment (Figure 10). Higher levels of ambient mROS accumulation were also observed in all cancer cell types tested compared to non-transformed cells. The emerging view is that activated metabolism and genetic instability under the control of oncogenic transformations cause increased ROS generation and decreased antioxidant systems in cancer cells. As a result, they harbor excess oxidative stress over normal cells. Such abnormal properties render cancer cells more prone than normal cells to cell damage induced by exogenous ROS-generating agents and such vulnerability can be exploited as a potential target for tumor-selective cell killing [38–41]. Our findings are closely matched with this concept, and provide insights into the molecular targets for the vulnerability. Since malfunction of either fission or fusion deeply impacts mitochondrial function and cell survival [24–27], the vulnerability of tumor cells to mitochondrial network collapse can contribute to tumor-selective cell killing.

In conclusion, we demonstrated for the first time that indirect AGP treatment can induce mitochondrial network collapse and cell death in tumor cells, but not in non-transformed cells. We further demonstrated that mROS accumulation plays a key role in these effects and that tumor cells are more vulnerable than non-transformed cells to mitochondrial oxidative stress. The present findings expand our previous study on TRAIL and reinforce the importance of mitochondrial network remodeling as a target for tumor-selective cancer treatment.

## MATERIALS AND METHODS

### Reagents

Soluble recombinant human TRAIL and mdivi-1 were obtained from Enzo Life Sciences (San Diego, CA). The general caspase inhibitor z-VAD-FMK and caspase-3/7-specific inhibitor z-DEVD-FMK were purchased from Merck Japan (Tokyo, Japan). The reagents were dissolved in dimethylsulfoxide and diluted with Hank's balanced salt solution (HBSS) (pH 7.4) to a final concentration of < 0.1% before use.

### Cell culture

The human melanoma cell lines (A375, A2058), human lung adenocarcinoma epithelial cell line (A549), and human osteosarcoma cell lines (MG63, SAOS-2, HOS) were obtained from the Health Science Research Resource Bank (Osaka, Japan). HDFs from the facial dermis were obtained from Cell Applications (San Diego, CA). All cell lines were cultured in DMEM (Sigma-Aldrich, St. Louis, MO) supplemented with 10% FBS (Sigma-Aldrich; FBS/DMEM) and streptomycin and penicillin (5000 Units/ml) and streptomycin (5000 μg/ml) (Pen Strep, Thermo Fisher Scientific Japan, Tokyo, Japan) in a 95% air/5% CO_2_ humidified atmosphere. Normal human epidermal melanocytes were obtained from Cascade Biologics (Portland, OR), and cultured in DermaLife Basal Medium supplemented with DermaLife M LifeFactors (Kurabo, Osaka, Japan). Cells were harvested by incubation with 0.25% trypsin-EDTA (Thermo Fisher Scientific Japan) for 5 min at 37°C.

### Generation of AGP

A non-thermal AGP jet was generated by an originally-developed LF plasma jet device. In this device, AGP was generated by an asymmetrical dielectric barrier discharge (DBD) with helium gas. The typical frequency was 20 kHz, with peak voltage of 8 kV, current of 20 mA, and helium flow rate of 300 ml/min. In the DBD region of the plasma source, nitrogen ions, nitrogen, oxygen and hydroxyl radicals were generated. These radicals were carried through a thin quartz tube and ejected into the atmosphere. AGP-activated medium (1 ml) was prepared by irradiating DMEM (1 ml) with AGP for 5 min, while control medium was exposed to helium gas without discharge.

### Cell-free ROS measurements

The ROS levels in AGP-activated medium were measured by the dROM tests (Wismerll Co. Ltd., Tokyo, Japan) using a FRAS4 Free Radical Analytical System (Wismerll Co. Ltd, Parma, Italy) according to the manufacturer's instructions. This device quantitatively measured the free radical levels in the medium and expressed the data in conventional arbitrary units, (Carr units), where 1 Carr unit was equal to 0.08 mg/dl H_2_O_2_ [42, 43].

### Cell proliferation, viability and cell death analyses

Cell proliferation was assessed using the cell counting reagent SF (Nacalai Tesque, Kyoto, Japan). Cells were seeded at a density of 2000 cells/well in 96-well plates (Coning, New York, NY) were incubated with the agents to be tested before 10 μl of cell counting reagent SF was added and further incubated for 1 h. The absorbances were measured at 450 nm using an ARVO MX microplate reader (Perkin Elmer, Waltham, MA). Cell viability was measured by the WST-8 assay, a colorimetric assay based on the formation of a water-soluble formazan product using a Cell Counting Kit (Dojindo, Kumamoto, Japan) as previously described [29]. Cell death was evaluated by fluorescence microscopy as previously described [29] with minor modifications. Briefly, cells (1 × 105 cells/well) were placed in an 8-well chambered coverglass (Thermo Fisher Scientific Japan) and treated with the agents to be tested for 24 h at 37°C in a 5% CO_2_ incubator. The cells were then stained with 4 μM each of calcein-AM and EthD-1 to label live and dead cells, respectively, using a commercially available kit (LIVE/DEAD^®^ Viability/Cytotoxicity Kit; Thermo Fisher Scientific Japan). Images were obtained and analyzed using an EVOS FL Cell Imaging System (Thermo Fisher Scientific Japan) equipped with a digital inverted microscope at × 300 magnification. Apoptotic cell death was quantitatively assessed by double-staining with FITC-conjugated annexin V and PI as previously described [30]. Briefly, after incubation with the agents to be tested for 24 h or 72 h in FBS/DMEM at 37°C, the cells were stained with FITC-conjugated annexin V and PI using a Phosphatidylserine Apoptosis Assay Kit (AAT Bioquest, Inc., Sunnyvale, CA) for 15 min at room temperature, washed with PBS, and resuspended in PBS. The stained cells were evaluated in a FACSCalibur^™^ (BD Biosciences, San Jose, CA) and analyzed using CellQuest^™^ software (BD Biosciences). Four cellular subpopulations evaluated: viable cells (annexin V^−^/PI^−^); early apoptotic cells (annexin V^+^/PI^−^); late apoptotic cells (annexin V^+^/PI^+^); and necrotic/damaged cells (annexin V^−^/PI^+^). Annexin V^+^ cells were considered to be apoptotic cells.

### Mitochondrial network imaging

The mitochondrial network was analyzed by staining with the mitochondria-targeting dye MitoTracker^®^ Red CMXRos (Thermo Fisher Scientific Japan) as previously described [28]. Briefly, cells in FBS/DMEM were placed at the density of 5 × 104 cells/300 μl/well in an 8-well chambered coverglass and treated with the agents to be tested for 24 h at 37°C in a 5% CO_2_ incubator. After removing of the medium by aspiration, the cells were washed with HBSS, and stained with 20 nM MitoTracker Red CMXRos in HBSS for 1 h at 37°C in the dark in a 5% CO_2_ incubator. The cells were then washed with and immersed in FluoroBrite^™^ DMEM (Thermo Fisher Scientific Japan). Images were obtained and analyzed using the EVOS FL Cell Imaging System at × 1200 magnification. For confocal microscopy imaging, samples were observed using an Airyscan laser scanning microscopy (LSM 880, Carl-Zeiss Microscopy Japan, Tokyo, Japan) equipped with a 63 ×, 1.20 n.a. oil-immersion objective (C-Apochromat; Carl Zeiss Microscopy Japan).

### mROS measurement

The mROS levels were measured using MitoSOX^™^ Red (Thermo Fisher Scientific Japan) [28] with minor modifications. Briefly, cells (1 × 106 cells/ml) suspended in HBSS were incubated with the agents to be tested for 4 h at 37°C, and then incubated with 5 μM MitoSOX^™^ Red for 15 min at 37°C for loading. The cells were washed, resuspended in HBSS, and measured for their florescence at 5–s intervals for up to 3 min in a microplate fluorescence reader (Fluoroskan ASCENT, Thermo Fisher Scientific Japan) with excitation and emission at 542 and 592 nm, respectively. The data were expressed as the total fluorescence intensity (FI) for 3 min.

### Immunoblotting

The levels of pDrp1 and Drp1 were assessed by immunoblotting. Briefly, after stimulating with the agents to be tested, cells were washed twice with ice-cold PBS, lysed with RIPA buffer (Nacalai Tesque, Kyoto, Japan) containing protease inhibitors, and homogenized by sonication using a Bioruptor UCD-250 (Cosmo Bio, Tokyo, Japan). After centrifugation, the resultant supernatant was measured for the protein content using a Pierce BCA Protein Assay Kit (Themo Fisher Scientific Japan) according to the manufacturer's instructions. After heating at 70°C for 10 min, samples (15–20 μg protein) were subjected to reducing SDS-PAGE using a NuPAGE^®^ Novex 4–12% Bis-Tris Gel (Thermo Fisher Scientific Japan) and transferred to iBlot Gel Transfer Stacks PVDF Regular (Thermo Fisher Scientific Japan). The resulting membranes were blocked with Blocking One (Nacalai Tesque) for 1 h at room temperature, washed with TBS containing 0.05% Tween 20 (TBS-T), and incubated with a primary antibody; anti-phosphor-Drp1 (Ser616; #3455) and anti-phosphor-Drp1 (Ser637; #4867S) (Cell Signaling Technology Japan, Tokyo, Japan) overnight at 4°C. After washing with TBS-T, the membranes were incubated with the secondary antibody (ECL^™^ Anti-rabbit IgG, horseradish peroxidase-linked whole antibody from donkey; GE Healthcare, Little Chalfont, UK) diluted in TBS-T for 1 h at room temperature. The signals were visualized with the ECL Prime Western Blotting Detection Reagent (GE Healthcare, Little Chalfont, UK) and analyzed using a Luminescent Image Analyzer LAS-4000 (Fuji Film, Tokyo, Japan). GAPDH was evaluated as a loading control.

### Statistical analysis

Data were analyzed by one-way ANOVA followed by a post-hoc Tukey test using add-in software for Excel 2012 for Windows (SSRI, Tokyo, Japan). All data were expressed as means ± SEM. Values of *P* < 0.05 were considered to indicate statistical significance.
